
JOURNAL INFORMATION
==============================
NLM Title Abbreviation: ScientificWorldJournal
Journal ID: ScientificWorldJournal
NLM Journal ID: 101131163
Journal ID: TSWJ

The Scientific World Journal

ISSN: 2356-6140
EISSN: 1537-744X
Publisher: Wiley

ARTICLE INFORMATION
==============================
PMCID: PMC6084247
PMID: 30147573
DOI: 10.1100/tsw.2001.200
Article ID: 646857
Article version: 1
Subjects: Short Report

Inhibition of Apoptosis by the BEACH Domain and WD Repeats of Gene lba that has Key Features of Both Protein Kinase A Anchor and chs1/beige Genes

Wang Jia-Wang
Howson Julie
Ghansah Tomar
Ninos John
Kerr William G. *
H. Lee Moffitt Cancer Center and Research Institute, University of South Florida, 12902 Magnolia Drive, Tampa, FL 33612, USA
*William G. Kerr: kerrw@moffitt.usf.edu
Publication date: 2001
Electronic publication date: 2001 Oct 26
Volume: 1
First page: 96
Last page: 96
Copyright: Copyright © 2001 Jia-Wang Wang et al.
Copyright year: 2001
License: This is an open access article distributed under the Creative Commons Attribution License, which permits unrestricted use, distribution, and reproduction in any medium, provided the original work is properly cited.
License URL: https://creativecommons.org/licenses/by/3.0/

==============================
Miami *Nature Biotechnology* Short Reports
*TheScientificWorld* (2001) 1 (S3), 96SR
ISSN 1532-2246; DOI 10.1100/tsw.2001.200

### INHIBITION OF APOPTOSIS BY THE BEACH DOMAIN AND WD REPEATS OF *lba* GENE THAT HAS KEY FEATURES OF BOTH PROTEIN KINASE A ANCHOR AND *CHS1/BEIGE* GENES

Jia-Wang Wang, Julie Howson, Tomar Ghansah, John Ninos, and William G. Kerr*

H. Lee Moffitt Cancer Center and Research Institute, University of South Florida, 12902 Magnolia Drive, Tampa, FL 33612
* kerrw@moffitt.usf.edu

**INTRODUCTION.** The *Chediak-Higashi* Syndrome patients suffer from a systematic immune deficiency including defects in T cell cytotoxicity, killing by NK cells, defective bactericidal activity and chemotaxis by granulocytes and monocytes. All cells in *beige* mice and CHS patients bear giant vesicles that cluster around the nucleus. The beige and chs1 genes have been cloned recently (1). However, despite these very provocative findings there still remains no direct evidence that CHS1 proteins associate with intracellular vesicles and thus a molecular explanation for defective vesicle trafficking and the protein missorting in these diseases is still sought. We have recently identified a novel beige-like gene in humans and mice that is induced by LPS stimulation in macrophages and B cells. This protein also contains a potential protein kinase A binding site and we thus designate it *lba*, LPS-responsive, beige-like and anchor gene.

**METHOD.** The *lba* gene was isolated and cloned by a gene-trapping strategy (2, 3) and 5'- and 3'- RACE. The BEACH domain and WD repeats were cloned together or separately into a pEGFP-N vector. RAW 264.7 cells were stably transfected. Apoptosis was induced by 0.5 μM staurosporine for 4, 8, 17 hr and assayed by Western blotting of whole cell lysates for the status of PARP (poly-ADP ribose polymerase) and CASPASE 3.

**RESULTS.** The novel gene *lba* has an orthologue in flies in addition to in mice and humans. It has key features of both *chs1*/*beige* and A kinase anchor genes. Expression of *lba* is induced following LPS stimulation of B cells and macrophages. The *lba* is expressed in many other tissues in the body and has three distinct mRNA isoforms that are differentially expressed in various tissues. Confocal and immuno-electronic microscopy indicate this protein associate with the trans-Golgi complex, lysosomes, endoplasmic reticulum and plasma membrane. The BEACH domain and WD repeats of *lba* gene can prevent apoptosis induced by staurosporine (Fig. 1). The cleavage of caspase 3 and PARP, two apoptotic markers, are inhibited by LBA fusion protein. The subcellular localization of the fusion proteins is changed by LPS stimulation and staurosporine induction.

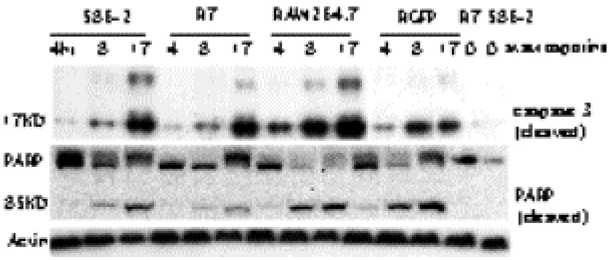

**Fig. 1.** Inhibition of apoptosis by LBA fusion proteins.

**DISCUSSION.** Homology analysis revealed that there is a new emerging gene family that contains a tandem arrangement of a BEACH domain and several WD repeats at their C-termini. These genes define a new protein family that we designate the WBW protein family. It includes *lba* gene in mice and humans and its orthologues in *C. elegans*, *Drosophila*, and its paralogues in diverse species ranging from yeast to human. Previous evidence suggested that WBW proteins are involved in intracellular vesicle trafficking. However, no direct evidence supporting this hypothesis for beige/CH1 genes has been obtained to date. Our results provide the first direct evidence that LBA and other WBW family proteins, CHS1/BG, FAN and LVSA etc., physically associate with the vesicular system and at least LBA directly involve in apoptosis, we propose, by binding to cytoplasmic tail of TNF-α or TNF-α-like receptors.

**REFERENCES.**

1. Kerr, W.G., Heller, M., and Herzenberg, L.A. (1996) Proc. Natl. Acad. Sci. U S A 93, 3947
2. Nagle, D.L., Karim, M.A., Woolf, E.A., Holmgren, L., et al. (1996) Nat. Genet.14, 307
3. Wang, J., Howson, J., and Kerr, W.G. (2000) Submitted
